# Compositionally Complex Alloys: Some Insights from Photoemission Spectroscopy

**DOI:** 10.3390/ma16041486

**Published:** 2023-02-10

**Authors:** Petar Pervan, Vesna Mikšić Trontl, Ignacio Alejandro Figueroa, Tonica Valla, Ivo Pletikosić, Emil Babić

**Affiliations:** 1 Institute of Physics, 10000 Zagreb, Croatia; 2 Institute for Materials Research—UNAM, Ciudad Universitaria Coyoacan, Mexico City 04510, Mexico; 3 Condensed Matter Physics and Materials Science Department, Brookhaven National Lab, Upton, NY 11973, USA; 4 Donostia International Physics Center, 20018 Donostia-San Sebastian, Spain; 5 Department of Physics, Princeton University, Princeton, NJ 08544, USA; 6 Department of Physics, Faculty of Science, University of Zagreb, 10000 Zagreb, Croatia

**Keywords:** compositionally complex alloys, high-entropy alloys, metallic glasses, amorphous alloys, electronic structure, electronic density of states—DOS, photoemission spectroscopy, UPS, XPS

## Abstract

Photoemission spectroscopy (PES) is an underrepresented part of current and past studies of compositionally complex alloys (CCA) such as high-entropy alloys (HEA) and their derivatives. PES studies are very important for understanding the electronic structure of materials, and are therefore essential in some cases for a correct description of the intrinsic properties of CCAs. Here, we present several examples showing the importance of PES. First, we show how the difference between the split-band structure and the common-band structure of the valence band (VB), observed by PES, can explain a range of properties of CCAs and alloys in general. A simple description of the band crossing in CCAs composed from the early and late transition metals showing a split band is discussed. We also demonstrate how a high-accuracy PES study can determine the variation in the density of states at the Fermi level as a function of Cu content in Ti-Zr-Nb-Ni-Cu metallic glasses. Finally, the first results of an attempt to single out the contributions of particular constituents in Cantor-type alloys to their VBs are presented. The basic principles of PES, the techniques employed in studies presented, and some issues associated with PES measurements are also described.

## 1. Introduction

The huge expansion of the research on compositionally complex alloys (CCA) such as the approximately equimolar, multicomponent high-entropy alloys (HEA) and their derivatives [1,2,3] and the corresponding amorphous alloys [4,5,6] has brought these systems to the forefront of research in materials science [7,8]. Since CCA design explores a previously unexplored middle section of the phase diagram, it makes an enormous number of new alloys available for research and possible technological applications [9,10]. One big advantage of this alloy design is that a broad compositional range in CCAs enables simple tuning of their properties by adjusting the concentrations of constituent elements and/or their composition. The opportunity to greatly advance our knowledge of these complex alloys (e.g., [11,12,13,14]) and achieve the industrial application of CCAs has sparked an intense research effort in the design, fabrication and study of these systems, which in turn has resulted in around a thousand new alloys, several thousands of research papers and dozens of reviews of the literature ([8,9,10,12,15,16,17,18,19]) and books ([20,21]).

Due to this intense research, great progress has been made. In some cases, a remarkable understanding of the properties of several technologically relevant CCAs, such as those with exceptional low- or high-temperature mechanical properties, has been demonstrated [9]. The CCAs show properties that are often superior to those of corresponding conventional alloys (CA) [8]. However, the basic understanding of CCAs is still quite limited, which is detrimental to their design and their application. We note that in addition to the intrinsic complexity of CCAs, the deficiencies in their current understanding also result from shortcomings in the previous research, as already noted in previously published papers [7,8,9,11,15,16,17,18,19,22,23,24,25,26,27,28,29]. Some examples of these caveats include the highly uneven distribution of research on CCAs regarding their composition [7], the frequently unjustified use of the rule of mixtures (ROM) for the calculation of their properties [22,23,24,25,26,27,28,29,30], the excessive use of mixing entropy to explain their formation and properties [11,15,16,17,18,19,22,23,24,25,26,27,28,29,30,31] and highly insufficient experimental studies of their electronic structure (ES) [11,22,23,28]. A lack of adequate insight into the ES, which in metallic systems determines almost all properties [32,33,34], is probably the main obstacle to the conceptual understanding of both crystalline (c-) and amorphous (a-) CCAs [11,22,23,28,30,35,36,37,38].

As noted in the previous publication [23], a rather good insight into the ES of CCAs (and alloys in general) can be obtained by the combination of the ab-initio (a-i) calculations (e.g., [9,12,13,33,36,37,38]), low-temperature specific heat measurements (LTSH), [11,23,26,27,28,30,35]) and PES studies [11,22,25,26,28,29]. PES on amorphous and polycrystalline alloys provides information on the electronic density of states (DOS) as a function of binding energy within the valence band (VB), while LTSH gives an accurate value of DOS at the Fermi energy *E_F_*, *N*(*E_F_*), but cannot distinguish contributions of alloying elements. Theory (a-i calculations) can in principle provide all these quantities, as well as the probable atomic structure (including chemical short-range order) and magnetic order. Still, the results of these studies are limited by the relatively small sample size, energy resolution and approximations involved in the calculations (e.g., [12]). It is therefore surprising that PES studies of CCAs were virtually nonexistent until about five years ago [22]. Actually, there was limited use of X-ray photoelectron spectroscopy of the core levels in recent studies of the surface oxidation and corrosion resistance of some CCAs [39,40]. However, these studies did not report the spectra of VBs of studied alloys [39,40], hence they cannot be used to explain other properties of these alloys. In our previous studies, we used PES of early (TE)–late (TL) transition metal types of a-CCAs to show the influence of particular TL = Co, Ni or Cu on their ES and related properties (such as mechanical, magnetic and superconducting properties) [11,22,25,26,28,29]. More recently [28], we started using PES in order to correlate the evolution of ES and magnetism in the Cantor type of alloys with variable Ni or Fe content [41,42]. Since these studies were devoted to specific alloys and few properties, they were not able to show the full potential of PES in advancing the understanding of ES and the properties of CCAs. That is why we decided to present more clearly (and in one place) in this paper the importance of PES studies in improving the current understanding of the intrinsic properties of CCAs. This was achieved by presenting four rather general problems in both a- and c-CCAs using results of detailed PES studies. 

In the following, we first give a brief description of the PES technique and issues associated with its sensitivity to the energy of the photons used and surface contamination. After that, we indicate typical problems concerning the electronic structure and properties of both c- and a-CCAs that can be solved using PES. These include the difference between the properties of CCAs exhibiting a split- or common-band shape of the VB, the application of PES to a study of the band crossing in CCAs showing split-band VBs, its application to the variation of *N*(*E_F_*) with alloying in a similar, early (TE)–late (TL) transition metal type of CCAs and the first results from an attempt to fit the line-shape of the common-band VB spectrum of the Cantor alloy [1].

## 2. Materials and Methods

### 2.1. Preparation, Processing and Characterization of Alloys

The materials, methods of preparation, processing and characterization of the samples and a technique of performing ultraviolet photoemission spectroscopy (UPS) measurements used for quinary Ti-Zr-Nb-Cu-Ni metallic glasses (MG) and the Cantor type of quinary Cr-Mn-Fe-Co-Ni, 3d transition metal alloys with the face-centered cubic structure have been described in some detail elsewhere [11,22,23,25,26,27,28,31,39,40,41,42,43]. For completeness, we briefly revisit some of the issues in those materials and then present a comprehensive PES study of the senary Al-Ti-Zr-Pd-Cu-Ni alloy.

The ingots of eight (TiZrNbCu)_1−x_Ni_x_ alloys (x = 0, 0.125, 0.15, 0.2, 0.25, 0.35, 0.43 and 0.5) [11,12,23,26,27,28] and eleven (TiZrNbNi)_1−x_Cu_x_ alloys (x = 0, 0.05, 0.12. 0.15, 0.2, 0.25, 0.32, 0.35, 0.43, 0.5 and 0.52) [25,28] were prepared from high-purity components (≥99.8 at. %) by arc melting in high-purity argon in the presence of a titanium getter. All ingots were remelted and flipped five times to ensure good mixing of components. Samples in a form of ribbons with a thickness of about 25 µm of each alloy were prepared by the melt-spinning molten alloy on the surface of a copper roller rotating at the speed of 25 m/s in a pure helium (He) atmosphere. As-cast ribbons were studied by X-ray diffraction (XRD) with Cu-Kα radiation [23,25,27,28]. The XRD patterns showed that all samples, except those with x = 0 and x = 0.52 Cu, were X-ray-amorphous (the atomic structure of amorphous ribbons was also investigated using synchrotron-based high-energy X-ray diffraction [11,25]). The samples that appeared X-ray-amorphous were further studied by differential scanning calorimetry and thermogravimetric analysis. Thermal measurements were performed up to 1600 K with a ramp rate of 0.67 K/s. These measurements provided the values of thermal parameters and confirmed the amorphous state of as-cast samples [11,23,25,27,28]. The characterization of the samples was completed with scanning electron microscopy (SEM) with energy-dispersive spectroscopy (EDS) capability to determine their actual composition and chemical homogeneity [11,25]. Elemental mapping was performed on three different areas of each sample.

The ingot of Al_0.5_TiZrPdCuNi alloy was prepared from high-purity components (≥99.8 at. %), following the same procedure as that described above for Ti-Zr-Nb-Ni-Cu alloys [44], to verify an earlier claim [45] that it can be prepared in either an amorphous or single-phase body-centered cubic (BCC) crystalline structure depending on the cooling rate from the molten state. Accordingly, both the melt-spun ribbons (melt spinning is reported to yield an amorphous single phase [45]) and a cylinder with a diameter of 1.5 mm obtained by the metallic mold-casting technique (this procedure was reported to yield a single-phase BCC alloy [45]) were prepared. However, our as-cast ribbons were X-ray-amorphous, but the cylinder had a multiphase structure, similar to that obtained in later attempts [46] to obtain a single-phase BCC structure. These amorphous ribbons were characterized in the same way as Ti-Zr-Nb-Ni-Cu ones and were also studied by XPS and magnetic measurements [44].

The ingots of five (CrMnCoNi)_1−x_Fe_x_ (x = 0, 0.1, 0.2, 0.3 and 0.5) and five (CrMnFeCo)_1−x_Ni_x_ (x = 0.2, 0.3, 0.5, 0.6 and 0.92) alloys were prepared from high-purity components (≥99.95 at. %) by using high-frequency induction melting in a water-cooled copper crucible under a pure He atmosphere, followed by suction casting into the shape of cylinders with 13 mm in diameter [31]. Subsequently, 2–3 mm thick slices of ingots wrapped in tantalum foils were annealed at 1373 K for six hours under a pure He atmosphere [31]. The annealed samples (still in a He atmosphere) were quickly cooled to room temperature. All annealed samples underwent a thorough characterization, which included XRD and SEM/EDS studies, as well as differential scanning calorimetry [28,31,39,40,41,42,43]. XRDs of all studied alloys exhibited four peaks corresponding to the single face-centered cubic (FCC) phase. The plate-like samples required for magnetization and UPS measurements were cold-rolled after casting down to the thickness of 0.5 mm and then annealed at 1373 K for 6 h under a pure He atmosphere.

### 2.2. Photoemission Spectroscopy

**Figure 1:**
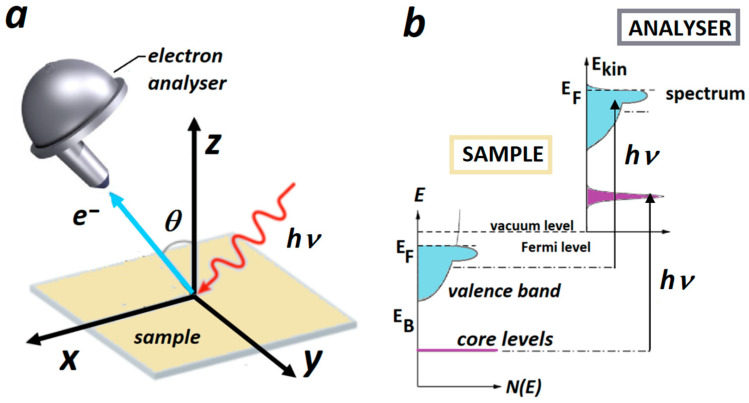
(**a**) Geometrical set-up of UPS showing incident light of energy hν exciting electrons in the sample. The electrons are collected and analyzed by the electron analyzer; (**b**) schematic diagram of the valence band and core levels being excited by the light of energy hν.

The principle of photoemission spectroscopy and its results for polycrystalline alloys are schematically presented in Figure 1. Photoemission spectroscopy—PES, also known as photoelectron spectroscopy, refers to the detection of emitted electrons excited in the solid by incident monochromatic light (Figure 1a). By measuring the kinetic energy of an outgoing electron and its emission angle, one can determine the binding energy of the electron and its corresponding momentum, revealing the band dispersions in crystalline solids [47,48]. Depending on the excitation energy of the incident light, electrons from a solid can be excited from the valence band and core levels (Figure 1b). According to the range of electron energies that can be accessed by the photoionization process, PES could be roughly divided to ultraviolet (UPS) and X-ray (XPS) photoelectron spectroscopy. UPS generally refers to the PES in the 5–100 eV photon energy range. The most common excitation energies used in laboratory environments are 21.2 eV and 40.8 eV corresponding to the He-I and He-II excitations. Al Kα and Mg Kα X-rays with the excitation energies of 1486.6 eV and 1253.6 eV are typically used in the laboratory environment for XPS. A continuum of photon energies from 5 eV to several tens of keV can be produced by synchrotron radiation for both UPS and XPS studies.

**Figure 2:**
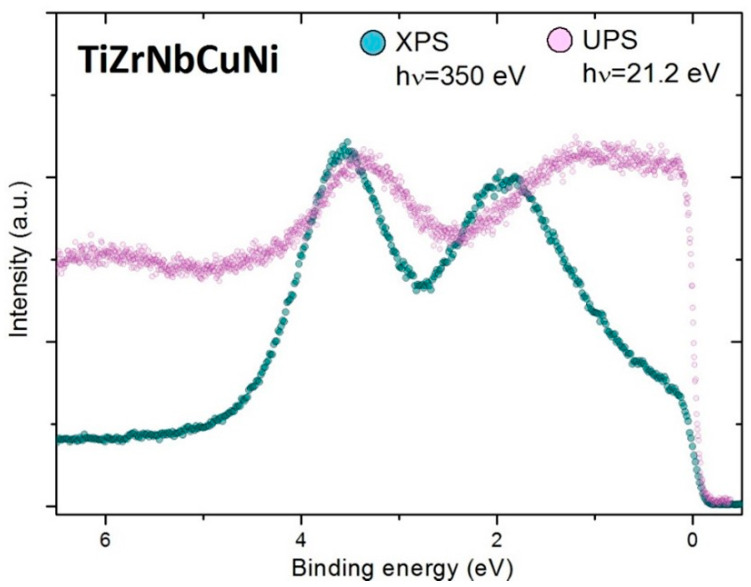
UPS and XPS spectra of TiZrNbCuNi alloy.

In this work, we present PES spectra obtained by 21.2 eV for UPS experiments and 350 eV (soft X-rays) for XPS experiments. In the amorphous HEA, the lack of long-range structural and compositional order is a suppressing factor for the band dispersion, as the electron momentum is ill-defined, and consequently, the intensity of PES spectra can only be qualitatively correlated to the total DOS. In polycrystalline samples, although the band dispersion is locally conserved, it cannot be resolved in standard photoemission experiments where the information is averaged over many different domains, and therefore blurred in ***k***—space. However, one has to bear in mind that the transition probability to excite an electron from a certain element depends on photon energy [47] (see Figure 2). For example, the excitation of a Ti 3d electron is roughly 150 times more probable with a 21.2 eV than with a 350 eV photon [49]. Similarly, excitations from Nb and Zr 4d are more probable than from Cu 3d for 21.2 eV excitation energy. On the other hand, at 350 eV photon energy, the major contribution to photoemission intensity in the NbZrTiNiCu alloys spectrum comes from Ni and Cu [49]. In other words, the shape of the photoemission spectrum generally depends on the energy of an incoming photon. 

**Figure 3:**
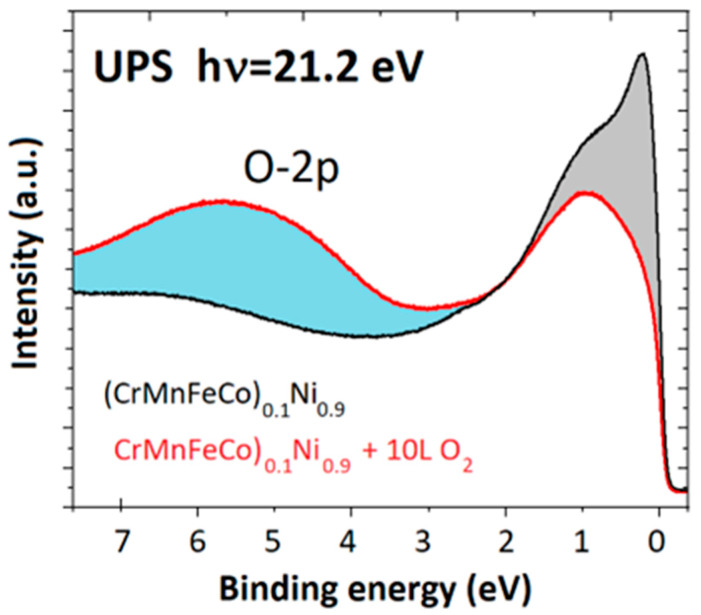
UPS of clean (CrMnFeCo)_0.1_Ni_0.9_ sample and thesame sample exposed to 10 L of O_2_ at room temperature.

The PES experiment is always performed in an ultrahigh vacuum (UHV), which ensures the passage of photoelectrons from the clean surface of a studied material to the analyzer. At the same time, the UHV prevents contamination of the sample. To obtain a clean sample surface, the sample was sputtered with 2 keV Ar+ ions at room temperature. The most common surface contamination on metallic samples was oxygen. The impact of oxygen on the UPS spectrum is clearly visible in Figure 3, which shows the spectrum of clean (CrMnFeCo)_0.1_Ni_0.9_ and the same sample exposed to 10 Langmuirs (L) of oxygen. The spectrum of the clean sample is dominated by the intensity between the 0 eV and 3 eV, which is associated with the valence band of the alloy.

The adsorbed oxygen shows up as an additional spectral maximum at around 6 eV. More importantly, there is an obvious change in the spectral intensity associated with the valence band of the alloy upon oxygen adsorption. A strong reduction in the spectral intensity between 0 eV and 1 eV with the most prominent drop at the Fermi level is a clear sign not only of the attenuation of the alloy’s signal, but also points to the possible chemical change induced in the probed region of the sample by the adsorbate.

The UPS spectra, presented in this work, were collected in laboratory conditions using a He-I excitation source to generate photons of 21.2 eV. The photoelectrons were collected and analyzed by Scienta SES100 hemispherical electron analyzer. The overall energy resolution in the experiments was about 25 meV. The XPS experiments were performed at 21-ID beamline of NSLS II synchrotron, Brookhaven National Laboratory, with 350 eV photon energy. The Scienta DA30 electron energy analyzer was used with 50 meV energy resolution.

It is important to point out that in addition to influencing the probability of excitation for different elements, the photon energy affects the mean free path of photoelectrons. From the electrons’ mean free path universal curve (e.g., [47,50]) we can estimate that photoemission using photons between 20 eV and 400 eV will probe electrons within 1 nm from the surface, meaning that any surface contamination would have a significant footprint in such photoemission experiments.

## 3. Results and Discussions

### 3.1. The Shape of Valence Band Spectrum—Information Value

**Figure 4:**
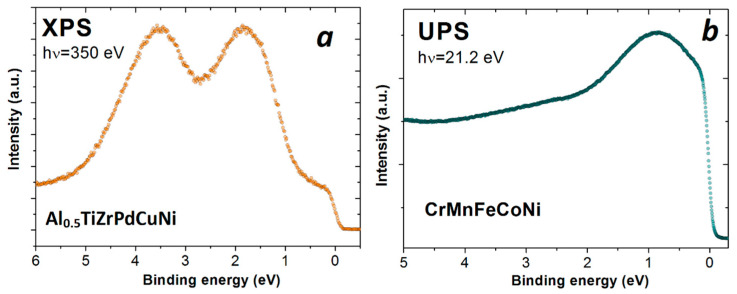
(**a**) XPS of Al_0.5_TiZrPdCuNi magnetic alloy; (**b**) UPS of FCC CrMnFeCoNi Cantor alloy.

The importance of PES as a technique that provides the DOS within the VB in understanding the basic properties of intertransition metal CCAs is best seen from a simple analysis of two spectra shown in Figure 4. The spectra were obtained from two very different alloys: a) Al_0.5_TiZrPdCuNi MG (XPS in Figure 4a), which is basically a TE-TL type of alloy (thus composed from transition metals that are far apart in transition metal series) and b) the Cantor alloy (UPS in Figure 4b), which is a CrMnFeCoNi solid solution with an FCC structure (thus composed from the neighbouring iron group metals). The corresponding spectra are also very different. The one corresponding to Al_0.5_TiZrPdCuNi MG shows a typical split-band structure of the VB [22,25,26,28,29], a knee close to *E_F_* and two pronounced maxima at higher binding energies. The one at around 1.8 eV corresponds to the AlTiZrPdNi group of metals, with the dominant contribution of Ni, while the intensity at around 3.5 eV comes mainly from Cu-3d-states. The spectrum of the Cantor alloy shows, within a single broad maximum around 0.8 eV below *E_F_*, a smooth variation in intensity (i.e., DOS) with energy. This suggests that neighboring elements tend to form a common band in which the contributions of d-states of constituent elements smoothly combine [36]. 

Split-band structures of VBs of TE-TL MGs and crystalline alloys were observed [51,52] soon after the discovery of these MGs [53]. They are characterized by the dominant contribution of the TE d-states (knees close to *E_F_* in Figure 2 and Figure 4a) to the *N*(*E_F_*) and by the d-states of TL at higher *E_B_* (peaks below *E_F_* in Figure 2 and Figure 4a). Thus, the effect of alloying with TL is approximately described by the dilution of a-TE(s) [54,55]. This simplifies the explanation of approximately linear variations of most properties of these MGs with TL content over a broad range of TL concentrations [56,57,58,59,60,61,62,63,64]. Further, *N*(*E_F_*) values of TE-rich alloys (determined from LTSH [54,65]) were higher than those of stable hexagonal close-packed (HCP) phases of Ti, Zr and Hf. They were, in fact, found to be close to those of cubic structures of TEs. This is important because high *N*(*E_F_*) in TE-rich MGs leads to enhanced superconductivity and magnetic susceptibility [54,55,56,57,61,62,63,64], but also to weaker interatomic bonding, and thus to lower elastic modula, strength, hardness and thermal stability [24,54,58,59,63,64]. The variations of the properties of TE-TL type of CCAs, both amorphous and crystalline, with TL content [11,22,23,25,26,27,28,29] are qualitatively the same as those in corresponding conventional alloys described above. This seems to imply that in TE-TL alloys, the effects of chemical complexity on the variation in their properties are of lesser importance [28]. An important consequence of a split-band structure of the VB of TE-TL alloys is that the approximate description of their ES in terms of valence electron count, VEC [11,17,18,28], is not appropriate. This is especially reflected in properties dependent on *N*(*E_F_*), such as the superconducting transition temperature, T_c_ [26,30,56,61]. Indeed, T_c_ of a single-phase BCC (ScZrNb)_1−x_(RhPd)_x_ HEAs [30], as well as that of (TiZrNbCu)_1−x_Ni_x_ [26] and Zr_1−x_(Ni or Cu)_x_ MGs [56], decreases with increasing VEC. This is in contrast to the increase in T_c_ with increasing VEC observed in the same range of VEC values in both crystalline and amorphous transition metal alloys composed from neighboring elements [30]. A simple explanation of this discrepancy, based on PES spectra of TE-TL alloys (such as those in Figure 2 and Figure 4) is that the presence of d-electrons of TLs (such as Ni, Cu and Pd) at *E_F_* as well as their contribution to *N*(*E_F_*) is small. Therefore, it is erroneous to use a total number of d-electrons of TLs in the calculation of VEC [11,18,30] for TE-TL alloys. Indeed, for Zr_1−x_Ni_x_ MGs [56], an effective VEC = 3 (which is consistent with the valency of Ni) instead of VEC = 10 for Ni in calculating the average values of VEC of these alloys brings the variation in their T_c_s with VEC [66] in perfect agreement with that in amorphous alloys composed from the neighboring TEs [30]. Before a brief discussion of the approximate description of alloys showing the common-band structure of the VB with average VEC, we briefly comment on the apparent similarity of spectra in Figure 2 and Figure 4, despite the somewhat different compositions of these alloys. Practically, the same positions of two peaks below *E_F_* in XPS spectra of both alloys imply that these peaks correspond to 3d-states of Ni (lower *E_B_*) and Cu (higher *E_B_*). The apparent absence of some similar features associated with 4d-states of Pd in a spectrum of Al_0.5_TiZrPdCuNi MG is probably due to the small photoionization cross section of 4d-states of Pd at these photon energies compared to that of 3d-states of Cu and Ni [67]. This explanation is consistent with a somewhat shallower minimum of intensity between two peaks in this alloy (Figure 4a) compared to that in TiZrNbCuNi (Figure 2). Simultaneously, a somewhat higher knee close to *E_F_* in Figure 2 compared to that in Figure 4a is probably due to higher total TE content in the TiZrNbCuNi alloy.

As is well known, VEC is a reasonable approximation for ES in the description of some properties of transition metal alloys showing a common-band structure of VBs (Figure 4b). Indeed, in such alloys composed of neighboring elements, VEC can be used for describing the variation in superconducting T_c_ on alloying [30] and to describe the variation in magnetic moments in FCC alloys of the near-neighbor magnetic 3d metals—the Slater–Pauling curve. VEC was also used to predict the formation of HEAs’ crystalline phase (BCC or FCC) based on 3d transition metals [68]. However, the experimental separation of the partial spectral weights of constituent elements contributing to the common-band structure of the PES spectrum of an alloy is very complex. Even in the case of a simple binary solid solution such as Cu-Pd alloys [67], proper determination of the partial spectral weights requires the study of spectra of several alloys spanning a full concentration range (0–100 at. %) and using different photon energies. The first attempt to locate the main contributions of some constituents in the UPS spectra of the Cantor-type Cr-Mn-Fe-Co-Ni alloys [42] will be presented at the end of this chapter.

### 3.2. Band Crossing or Ideal Solution Behavior

An interesting feature of TE-TL alloys associated with their split band structure of DOS is the band crossing, which occurs at elevated TL contents [26,28,69,70,71]. This phenomenon was first reported for Zr-TL (TL = Fe, Co, Ni) MGs, where it showed up as the change in sign of both the Hall coefficient, *R*_H_ and the thermopower, *S* [69,70,71]. DOS for the Zr 4d states in these alloys has a broad peak at high energies (low binding energy, *E_B_*) and only a relatively small component at low energies (high *E_B_*, well below *E_F_*). The DOS for the TL component, the solute, has a peak at low energies (higher binding energies, *E_B_*) and only a small component at high energies [69]. (This shift between the positions of the maxima in DOS for Zr and TL, respectively, shows up in a split-band structure of their photoemission spectra [51,52].) The *E_F_* for Zr-rich alloys lies in the upper peak, “the Zr 4d band”. When the concentration (*x*) of TL increases, the relative magnitudes of the DOS in different energy ranges change and *E_F_* shifts towards the lower peak, the d-band of TL solute. Thus, at a certain crossover concentration *x_c_*, the *E_F_* of Fe, Co and Ni solutes is expected to shift from the Zr d band into the TL solute d band_._ [69,71]. The values for *x*_c_ of Zr-TL alloys may be obtained from the following formula [69]:

(1) $$n_{TL}x+n_{Zr}\left(1-x\right)=10x$$

Here, *n* stands for the number of d electrons of a particular atom. By using *n* = 2, 7, 8 and 9 for Zr, Fe, Co and Ni, respectively, this formula predicted the exact values of *x*_c_, at which thermopower *S* = 0 was observed in the Zr-TL MGs [69,70]. Clearly, this band crossing should occur in all properties of TE-TL alloys that are directly related to ES [69]. These are the Sommerfeld coefficient in the LTSH, the magnetic susceptibility, and of course PES. However, its effect on *S* is probably particularly instructive. Equation (2) is known as the Mott expression (e.g., [54]) for thermopower:

(2) $$S\left(T\right)=\frac{\pi^{2}k_{B}^{2}T}{3\left|e\right|}{\left(\frac{\partial ln\rho \left(E\right)}{\partial E}\right)}_{E_{F}}$$

where *e* is the charge of the electron, *k_B_* is the Boltzmann constant and *ρ* is the electrical resistivity. The Mott equation describes S quite well for liquid and amorphous transition metal alloys [54,72,73], assuming that the scattering of *s*- and *p*-electrons into empty *d* states dominates *ρ*. In other words, *ρ* is proportional to *N*(*E_F_*), which leads via Equation (2) to *S*$\sim {\left(\frac{\partial lnN\left(E\right)}{\partial E}\right)}_{E_{F}}$. Accordingly, *S*(*x*_c_) = 0 means that at the crossover concentration DOS at *E_F_* passes through a minimum; thus, the slope of variation of *N*(*E*) with *E* close to *E_F_* changes on crossing *x*_c_. This makes studies of band crossing by using PES particularly interesting. To our knowledge, no such study has been performed yet.

**Figure 5:**
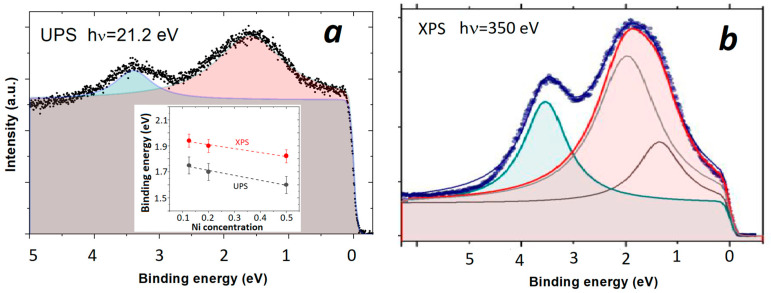
(**a**) UPS and (**b**) XPS of (TiZrNbCu)_0.5_Ni_0.5_ alloy. The inset shows the shift of Ni-related spectral maximum with Ni concentration.

The preliminary study showed that at the edge of the glass-forming range, *x* = 0.5, *R*_H_ of (TiZrNbCu)_1−x_Ni_x_ MGs start to decrease [26], indicating the vicinity of *x*_c_. Since we were unable to obtain fully amorphous alloys with *x* > 0.5, here we show only how approaching the *x*_c_ value affects the position and width of the maximum of the Ni *d* states studied with UPS and XPS (Figure 2 and Figure 5) (a recent finding that in (TiZrNbCu)_1−x_Co_x_ MGs, RH changes sign within their glass-forming range [74] provides a unique opportunity to study the band crossing in a chemically complex TE-TL MGs). To estimate the peak positions, the UPS and XPS spectra of (TiZrNbCu)_0.5_Ni_0.5_ (see Figure 5) were fitted using a Lorentzian line shape superimposed on the constant background terminated at the *E_B_* = 0 with Fermi-Dirac distribution. Notice that the spectral maximum at around 1.5 eV (see Figure 5b) related to the TiZrNbNi group was fitted with two Lorentzians reflecting the structure of the Ni-dominated [75] common band of TiZrNbNi. Note that both UPS and XPS spectra show that the Ni 3d peak shifts towards *E_F_* (or vice versa) with increasing Ni content from *x* = 0.125 to 0.5 (inset to Figure 5a). However, the positions of these peaks for the same *x* obtained from UPS or XPS measurements are not identical; peaks determined from XPS were found at 0.2 eV or higher binding energies when compared to UPS measurements (inset to Figure 5a). This difference, probably associated with an enhancement of the photoionization cross section of TEs at low photon energies, emphasizes the importance of performing both the XPS and UPS measurements when studying ES of TE-TL CCAs [28]. In the present case, where we want to find out how the contribution of the Ni d-state to the DOS at *E_F_* evolves with increasing *x,* the XPS measurements are preferred over UPS because they show in more detail the peak associated with Ni (Figure 5a and Figure 5b, respectively). From the small shift of *E_B_* towards *E_F_* of about 0.14 eV (less than 10% of initial *E_B_* throughout the explored concentration range) obtained from both UPS and XPS results (inset to Figure 5a), one may erroneously conclude that Ni d-states, even at *x* = 0.5, contribute only a little to the DOS at *E_F_*. However, an analysis of the evolution of the full width at the half maximum of a peak corresponding to d-states of Ni in the XPS spectrum with *x* shows that this width increases from about 1 eV for *x* = 0.125 to 1.62 eV or 62% at *x* = 0.5. Thus, the increase in the contribution of Ni d-states to *N*(*E_F_*) on increasing *x* in these alloys is largely due to the change in the width of the “Ni d band“ with increasing Ni content (the increase in the contribution of Ni d-states to *N*(*E_F_*) at elevated Ni contents was confirmed in LTSH measurements [11,26,27]). Thus, the studies of band crossing in TE-TL alloys should comprise XPS measurements. However, if subtle changes in *N*(*E_F_*) in alloying of the TE-containing alloys are to be studied, it is advantageous to use the enhanced photoionization cross section of TEs at low photon energies, as described in our next example.

**Figure 6:**
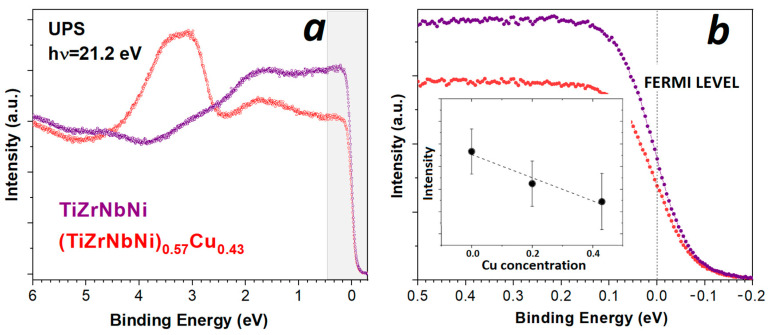
UPS of TiZrNbNi and (TiZrNbNi)_0.57_Cu_0.43_ showing (**a**) valence band region; (**b**) expanded region (grey area in (**a**)) around Fermi level, with inset indicating the change in the spectral intensity at the Fermi level for different concentrations of Cu.

As was recently emphasized, despite being composed of the same constituents as (TiZrNbCu)_1−x_Ni_x_ ones [11,26,27,28], (TiZrNbNi)_1−x_Cu_x_ MGs show very different behavior [25,28]. These MGs, like the binary TE-Cu ones [54], show a linear, Vegard’s law [76] (or rule of mixtures) variation in most of their properties with Cu content *x*. Moreover, their properties extrapolate to those of a pure FCC Cu for *x* = 1 [25,54]. The ideal solution behavior of all TE-Cu MGs is astonishing. Namely, the parameters of TE-Cu alloys do not meet the requirements for Vegard´s law behavior in crystalline solid solutions, such as the small difference in atomic size, similar chemical properties (such as electronegativity and oxidation states) and the same or similar stable crystalline structure of constituent elements [54,76,77]. Accordingly, it appears that amorphous atomic structure promotes variations of Cu MG properties according to Vegard’s law (Vegard’s law variation in atomic volumes is a synonym for simple additivity of atomic volumes of the constituents). In support of that claim, we note [25,54] that Vegard’s law variation in atomic volumes can be attributed to the approximate cancellation of the negative volume change associated with strong interatomic bonding between Cu and TEs (which manifests in a large negative heat of mixing [25,28]) and a positive volume change due to the lower density of a random atomic packing as compared to a close-packed corresponding crystalline structure. As a result, there is no band crossing for *x* < 1 in these MGs. Their Cu d band remains well below *E_F_* for all values of *x* (Figure 2 and Figure 6, and [25,28]), throughout the glass-forming range of these alloys. This results in a linear decrease in *N*(*E_F_*) with increasing x [54]. Thus, the LTSH measurements in binary TE-Cu MGs can be used to estimate *N*(*E_F_*) both for amorphous TEs and Cu [54,65].

To single out the contributions of each TE (Ti, Zr and Nb) to *N*(*E_F_*) in our quinary MGs, one has to measure the LTSH of several MGs with different concentrations of each TE [28,78]. However, the study of photoemission intensity variation at *E_F_*, with Cu content in such alloys, can provide insight into the accuracy and energy resolution of the PES measurements used. This is illustrated in Figure 6a,b, in which we compare the UPS spectra for MGs with *x* = 0 and 0.43 (Figure 6a). Figure 6b clearly shows that the intensity decreases at *E_F_* with increasing *x.* Further, this decrease in intensity with Cu content is approximately linear (inset to Figure 6b), and the slope of this decrease is like that of the Sommerfeld coefficient (and thus also of *N*(*E_F_*)) obtained from the LTSH measurements of the same alloys (Figure 13 in [28]). This demonstrates the high quality and great usefulness of UPS measurements in correlating spectroscopic and specific heat results and in determining the intrinsic properties of these alloys.

### 3.3. Effects of Some Constituents on Spectra of Cantor-Type Alloys

**Figure 7:**
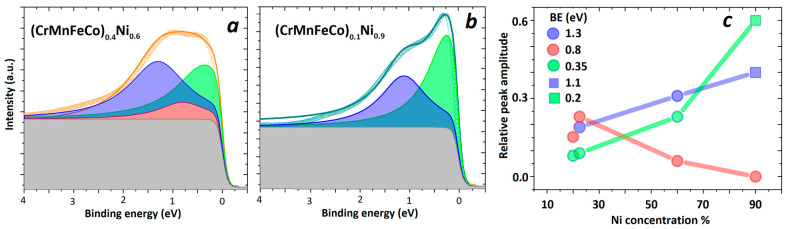
UPS of (CrMnFeCo)_1−x_Ni_x_ and corresponding Lorentzian peaks used to deconvolute the spectrum for (**a**) *x* = 0.6 (**b**) *x* = 0.9. (**c**) Plot of the relative peak amplitude as a function of Ni concentration with binding energy (BE) of a corresponding peak indicated.

The Cantor alloy (CrMnFeCoNi), the archetypal HEA [1], which exhibits a common band feature in UPS (Figure 4b), has been studied more than any other CCA [9,12,19], primarily due to its intriguing mechanical properties, such as its outstanding strength and fatigue resistance at low temperatures [8,9,19]. There are also numerous theoretical studies of the ES [12,13,36,38,79] and magnetic properties (e.g., [28,80,81]) of this alloy. All theoretical studies predict a ferromagnetic ground state for the Cantor alloy. However, the corresponding values of the Curie temperature, *T*_C_ and the average magnetic moment *m* depend on the calculation method used (e.g., *T*_C_ = 23 K and *m* = 0.39 µ_B_ was calculated in [38]). These same—but experimentally determined—parameters also show a large scatter, which is most likely a consequence of the difficulties in achieving a homogeneous distribution of the constituents in the solid solution due to their different bonding tendencies to other constituents [39,40,41,42,43]. The corresponding scatter in the values of the magnetic parameters of the Cantor alloy, obtained by different calculations, is probably associated with the intricacies of these calculations [13]. A recent study of the evolution of ES and magnetism on the transition from the Cantor alloy to a pure Ni in FCC (CrMnFeCo)_1−x_ Ni_x_ alloys [28], which probably solved the problem of inhomogeneities, revealed a nonmagnetic ground state of the Cantor alloy (*x* = 0.2) and the onset of a long-range ferromagnetic order for *x* > 0.3. In alloys with *x* ≥ 0.5, T_C_ and m values smoothly approach the values of pure Ni. A parallel ES study using UPS revealed that their respective spectra evolve from one with a shape similar to the calculated DOS of the Cantor alloy [38] to one similar to the DOS of pure Ni in the alloy with *x* = 0.92. However, the spectra retained a common band shape for all values of *x.* We are unaware of ES calculations for these alloys that include their partial constituent DOSs, thus preventing any deeper insight into the evolution of their DOS and associated UPS spectra. Motivated by the lack of any insight into the structure of DOS-related spectra, we made an attempt to resolve the main contributions of the observed UPS spectra and their evolution with the Ni content, *x*. The employed method and its main result are illustrated in Figure 7. As shown in Figure 7a, the UPS spectrum is fitted with Lorentzians superimposed on a constant background, all terminated by Fermi–Dirac distribution. 

For the Ni concentration, x≤0.6, three Lorentzians were required, while the spectrum corresponding to *x* = 0.9 was successfully fitted with only two (Figure 7b). The same procedure was applied to all samples having variable Ni and Fe content ((CrMnCoNi)_1−x_Fe_x_ [28]), altogether seven alloys. The spectra for all these alloys were fitted quite well, with three Lorentzians having maxima at approximately the same values of *E_B_* (about 0.35, 0.8 and 1.3 eV, Figure 7a). It was only for (CrMnFeCo)_0.1_Ni_0.9_ that we noticed a binding energy shift to lower energies (from 0.35 eV to 0.2 eV and from 1.3 eV to 1.1 eV). 

To help the understanding of the magnetic properties of these alloys, we were particularly interested in the evolution of the contributions of ferromagnetic constituents Ni, Co and Fe to the measured UPS spectra. We plotted the amplitudes of these Lorentzians (normalized to the background intensity) vs. the contents of Ni, Co or Fe. As seen from Figure 7c, the peak amplitude vs. Ni content plot revealed a positive correlation of the Lorentzian centered around 0.35 (0.2) and 1.3 (1.1) eV below *E_F_* with the Ni content and a (weakly) negative one with that centered around 0.8 eV. The results in Figure 7c probably indicate that the contribution of Ni d-states in the UPS spectra is strong between 1.3 and 1.1 eV but very strong between 0.35 and 0.2 eV, as judged from the rapid increase in their contributions to the spectra with increasing Ni content. This result is consistent with the evolution of the magnetic properties of these alloys towards the characteristic of pure FCC Ni [28]. Full results of this research, accompanied by the corresponding magnetic properties, will be published in the forthcoming publication [82].

## 4. Conclusions

In this work, we aimed to emphasize the importance of photoemission spectroscopy in the studies of the electronic structure of compositionally complex alloys by using selected examples from the recent research of both complex crystalline and amorphous alloy systems. 

Despite all possible shortcomings arising from the limited information that can be obtained from amorphous materials by this technique, our examples have shown that such electronic structure information is still sufficient to explain some previously unexplained or misexplained phenomena in alloys composed from early and late transition metals. These are, for example, variations in the superconducting transition temperatures with the valence electron count or the enhancement of the thermal stability, strength and hardness with increasing mixing entropy in multicomponent, transition metal glasses. We have demonstrated that these phenomena can be simply explained by the split-band structure of their photoemission spectra, reflecting the corresponding variation in the density of states in these alloys. Furthermore, we note that photoemission spectroscopy can be used to obtain direct insight into band crossing in early and late transition metal alloys. In that context, the influence of photon energy on the position and width of the peaks in the photoemission spectrum has been discussed. 

We also demonstrated how UPS spectra can be used to obtain qualitative but reliable information about the variation in the density of states at the Fermi level upon alloying.

In the final example, we presented the first results of an attempt to deconvolve the UPS spectra of face-centered cubic Cr-Mn-Fe-Co-Ni alloys with variable Ni content. Due to the smooth intensity variation with binding energy, reflecting the formation of a common band upon alloying of these 3d elements, it was impossible to assign part of the spectrum to one constituent element or to several constituent elements. Nevertheless, we were able to associate Ni 3d states with the spectral intensity increase at distinct binding energies. We hope that our work will stimulate the use of photoemission spectroscopy in the study of composite complex alloys, which will greatly improve the understanding of the underlying physics of these materials.

## Data Availability

Reasonable requests for data can be addressed to V.M.T.

## Abbreviations

a-CCA	amorphous CCA
BCC	body-centered cubic (crystalline structure)
CA	conventional alloys
CCA	compositionally complex alloys
c-CCA	crystalline CCA
DOS	density of states
*E_B_*	binding energy
EDS	energy-dispersive spectroscopy
ES	electronic structure
FCC	face-centered cubic (crystalline structure)
HCP	hexagonal close-packed (crystalline structure)
HEA	high-entropy alloy
L	Langmuirs
LTSH	low-temperature specific heat measurements
MG	metallic glasses
PES	photoemission spectroscopy
ROM	rule of mixtures
SEM	scanning electron microscopy
TE	early transition metal
TL	late transition metal
UHV	ultrahigh vacuum
UPS	ultraviolet photoemission spectroscopy
VB	valence band
VEC	valence electron count
XPS	X-ray photoemission spectroscopy
XRD	X-ray diffraction
